# Comparative Study on Injection Molding and Performance of Glass Fiber-Reinforced PET and PA6 Thermoplastic Insulators

**DOI:** 10.3390/ma19091729

**Published:** 2026-04-24

**Authors:** Yao Wang, Yuliang Fu, Xiaofei Chen, Zehao Zhang, Weiqi Qin

**Affiliations:** 1 Tai’an Taishan High Voltage Switchgear Co., Ltd., No. 50 Boyang Road, Taishan District, Tai’an 271000, China; wangyao0516@163.com; 2 Department of Electrical Engineering, Tsinghua University, Beijing 100084, China; yfu34@ncsu.edu (Y.F.); zzh1504399834@gmail.com (Z.Z.); 3 Department of Mechanical and Electrical Engineering, Beijing Information Science and Technology University, Beijing 100192, China; 18954855649@163.com

**Keywords:** glass fiber-reinforced PET insulators, glass fiber-reinforced PA6 insulators, Moldflow simulation, ANSYS, COMSOL, hydraulic pressure testing, finite element analysis, injection molding process

## Abstract

In ultra-high-voltage GIS and GIL systems, epoxy resin insulators are still the mainstream choice. However, as a thermosetting material, epoxy resin is difficult to recycle after disposal, which limits its environmental benefits. Thermoplastic insulators, due to their recyclability, are potential alternatives. This study focuses on 30% glass fiber-reinforced PET and PA6 materials. Their injection molding behavior, hydraulic pressure performance, and insulation performance were systematically analyzed using Moldflow, ANSYS, and COMSOL, respectively. For injection molding, Moldflow simulations were conducted for filling, packing, and cooling stages. Melt temperature was varied from 260 to –310 °C (PET) and 250–300 °C (PA6), while mold temperature was varied from 80 to –130 °C (PET) and 70–120 °C (PA6). An optimization objective function, Y = Δp/20 + Δx/0.5 + Δs/1.8, was developed to determine optimal processing parameters. Based on this function, the optimal parameters identified are: PET at 290 °C melt temperature and 120 °C mold temperature; PA6 at 250 °C melt temperature and 70 °C mold temperature. For hydraulic testing, Moldflow–ANSYS coupled simulations were performed under 2.4 MPa pressure with the compliance criteria of bulk stress < 90 MPa and insert-contact stress < 20 MPa. PA6 passed within a processing window of melt temperature < 270 °C and mold temperature < 120 °C. PET failed under all tested conditions, with insert-contact stress ranging from 24.25 to 27.55 MPa, consistently exceeding the 20 MPa threshold. In terms of insulation performance, this paper utilizes COMSOL to study the electric field distribution of thermoplastic insulators in SF_6_ GIS/GIL and provides optimization suggestions for insulator geometry design. This study systematically compares the injection molding processes and hydraulic pressure performance of PET and PA6 thermoplastic insulators. These results provide important process insights and design guidance for evaluating thermoplastic materials as potential alternatives to epoxy resin in GIS/GIL applications.

## 1. Introduction

As one of the most widely used insulating devices in power systems, the vast majority of insulators are currently made from epoxy resin-based composite materials [1]. Conventional epoxy resin waste faces significant recycling challenges. While research exists on chemical degradation, thermal decomposition, and supercritical fluid treatment for epoxy resin recycling, common disposal methods include incineration and deep burial, both of which cause significant environmental pollution [2,3]. The difficulty of recycling increases the operational cost of epoxy resin insulators and considerably undermines their environmental benefits. Based on this, developing environmentally friendly and recyclable insulating materials to replace existing thermosetting epoxy resins is of great importance.

Y. C. Zhang et al. demonstrated the feasibility of thermoplastic materials as environmentally friendly alternatives due to their recyclability through melting after disposal [3]. Currently, Volk et al. and Bessede et al. have explored the application potential of various thermoplastic materials, such as polyesters and polyamides, in insulators [4,5]. Studies by Z. Liang et al. indicated that glass fiber-reinforced PET (polyethylene terephthalate) exhibits a performance comparable to epoxy resin in several aspects and is considered one of the most promising alternative materials. For instance, Z. Liang et al. reported that incorporating short glass fibers can increase the tensile strength and dielectric strength of PET by approximately 50% and 25.4%, respectively [6]. However, in his research on thick-walled injection-molded products, Liu Sizhe found that configuring injection molding process parameters for PET material is challenging, making it difficult to obtain high-quality finished products [7]. Similarly, studies by Chen Zhi et al. on thick-walled high-density polyethylene products revealed the significant impact of process parameters on shrinkage and defects, further confirming the challenges in manufacturing thick-walled components [8]. A review of the existing literature reveals several key limitations in the current research. Liu Sizhe demonstrated that for ultra-thick-walled parts, conventional process parameter optimization alone is insufficient to eliminate shrinkage cavities [7]. Volk et al. highlighted that the performance of thermoplastic composites is critically dependent on raw material quality and processing parameters. Bessede et al. pointed out that no specific standards currently exist for thermoplastic-based insulators. Zhang et al. noted that systematic evaluation frameworks for selecting suitable thermoplastics remain lacking [3,4,5]. These identified limitations collectively demonstrate that while thermoplastic materials show promise for insulator applications, systematic research on manufacturing process optimization, particularly for thick-walled GIS/GIL components, remains limited. Most existing trial productions are small-scale experiments, and Liu Sizhe pointed out the lack of systematic parameter studies and quantitative stress analysis. Therefore, the manufacturing process for thermoplastic insulators still requires in-depth exploration [7].

To address the current lack of systematic research on the manufacturing process of thermoplastic insulators, this paper investigates the injection molding processes and comprehensive performance of PET and PA6 materials through integrated simulations using Autodesk Moldflow Insight 2023, ANSYS Workbench 2023 R1, and COMSOL Multiphysics 6.1. Notably, this study is simulation-based and focuses on establishing theoretical foundations and parameter optimization guidelines for future experimental validation. It systematically reveals the influence of key process parameters on the stress deformation of injection-molded parts and their performance in hydraulic pressure tests. Given the significant technical hurdles encountered in current PET trial productions, including difficulties in parameter configuration and inconsistent product quality. By comparing the processing characteristics and insulation design requirements of the two materials, the study demonstrates the advantages of PA6 over PET in terms of process feasibility and performance potential as an alternative. Furthermore, it provides a systematic theoretical analysis of the injection molding process for thermoplastic insulators.

## 2. Research Methods

### 2.1. Material Selection and Model Establishment

Current trial productions of thermoplastic insulators predominantly focus on PET materials [4,5]. However, PET melt exhibits poor flowability, requiring higher temperatures and injection pressures during the molding process. Its narrow adjustable range of processing parameters and significant molding difficulties pose challenges. In contrast, PA6 melt offers good flowability and a lower melting point, with a wider adjustable range of processing parameters, making the injection molding process relatively less challenging. Given the significant technical hurdles encountered in current PET trial productions, the potential of PA6 as an alternative insulating material warrants further investigation [3]. Specifically, the material grades selected are PETRA^®^ 130FR BK112 (BASF Corporation, Florham Park, NJ, USA) and Ultramid^®^ B3EG6 (BASF; data source: Material Data Center, Altair Engineering GmbH), both containing 30% glass fiber reinforcement (E-glass type) A comparison of the electrical and thermal properties of the two materials is presented in Table 1 and Table 2 [9,10].

It can be observed that PA6 exhibits the best performance in terms of dielectric strength and dielectric loss, while PET closely approaches epoxy resin in volume resistivity. Overall, the electrical properties of both materials are superior to those of epoxy resin.

It can be observed that both PET and PA6 possess higher heat deflection temperatures, making them suitable for high-temperature operating environments. PA6 has a relatively lower glass transition temperature. This lower Tg implies that PA6 insulators may experience greater molecular mobility and potential dimensional changes during normal operation, which could affect their long-term mechanical stability and creep resistance. However, the presence of 30% glass fiber reinforcement helps maintain structural integrity above Tg, and the heat deflection temperature of 210 °C ensures the short-term ability to withstand high -temperatures. This trade-off between processing advantages (broader temperature window) and service performance (potential Tg-related concerns) must be carefully considered in material selection to establish a broader processing temperature window.

**Figure 1:**
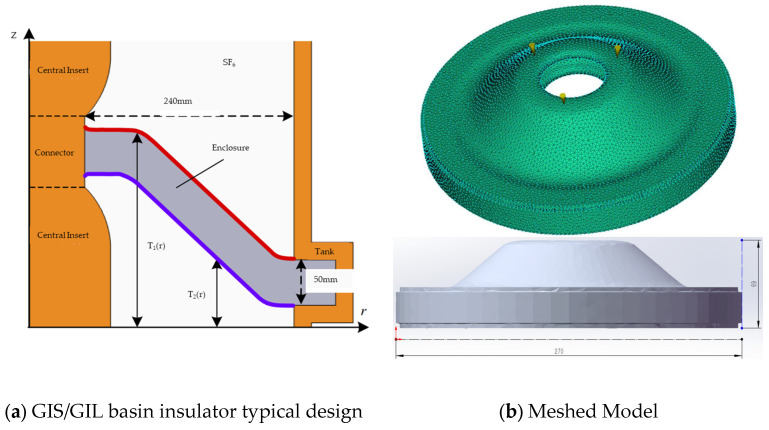
GIS/GIL basin insulator typical design and meshed model.

This work references the general design of basin-type insulators used in GIS/GIL [11]. The model was meshed using a 3D tetrahedral element type with a global element size of 4 mm, selected based on the thick-walled nature of the part and the focus on stress and warpage analysis. The final mesh consists of 400,203 tetrahedral elements. The insulator has a diameter of 270 mm and a height of 69 mm, with inner and outer annular vertical surfaces designed to interface with metal inserts. The central annular portion connects to the insert carrying the high-voltage conductor, while the outer edge connects to the enclosure. The basin-type insulator geometry was selected as a representative configuration for GIS/GIL applications because basin-type insulators are widely used in gas-insulated systems [11]. This geometry presents characteristic challenges for injection molding, including non-uniform wall thickness, complex curvature, and critical interfaces with metallic inserts, making it an appropriate test case for evaluating the manufacturability of thermoplastic materials. A geometric model of the thermoplastic basin-type insulator employed for simulation is shown in Figure 1.

### 2.2. Simulation Procedure and Parameter Settings

This study focuses on the entire injection molding process from injection to cooling and mold opening, with particular attention to the deformation and stress distribution of the molded part. Therefore, the analysis sequence selected in Moldflow is Fill + Pack + Warp. The software version used is Autodesk Moldflow Insight 2023. The investigation primarily targets two key parameters, melt temperature and mold temperature, as they are most directly related to warpage deformation and residual stress.

Based on the recommended parameter ranges and with further extension, the data scope for this study was established. A single injection gate was designed at the center of the insulator’s bottom surface, utilizing a three-gate symmetrical sprue system to ensure balanced melt flow. The runner system was designed with circular cross-sections of 8 mm diameter for the main runner and 6 mm for branch runners. The final process parameter values or ranges used in the simulation are shown in Table 3.

As shown in Table 3, the processing temperature range of PA6 is generally lower than that of PET, indicating greater flexibility in its process control. Based on the Moldflow simulation results, a coupled simulation of ANSYS and Moldflow was conducted. The simulation workflow involved exporting the volumetric mesh and residual stress tensor from Moldflow to ANSYS Workbench 2023 via the Moldflow–ANSYS interface. The geometric mesh obtained from Moldflow was imported into ANSYS, and the simulated stress tensor was also imported as initial stress. According to the hydraulic test conditions, a fixed constraint was applied to the contact surface between the molded part and the insert, while a normal pressure of 2.4 MPa was applied to other surfaces for static simulation. The 2.4 MPa hydraulic pressure test condition follows the standard requirements for GIS/GIL insulator qualification. According to the design practice for high-voltage gas-insulated equipment, the hydraulic pressure test for basin-type insulators typically applies a pressure of 2.4 MPa, which corresponds to approximately three times the design pressure (0.8 MPa) as specified in relevant standards. The burst pressure during hydraulic testing should exceed 2.4 MPa to verify its mechanical integrity under extreme conditions. The compliance criteria require that the von Mises stress within the insulator should not exceed the tensile failure stress of the composite material (typically 70 MPa), and the stress at the interface between the insulator and the metal insert should not exceed the bonding tensile strength (typically 20–25 MPa). These criteria are adopted as reference standards for evaluating the mechanical performance of thermoplastic insulators in this study [12]. For the electric field distribution of the insulator in the gas-insulated system, electrostatic field simulation was performed using COMSOL. A two-dimensional axisymmetric model was established to represent the typical GIS/GIL configuration, including the basin-type insulator, the central high-voltage conductor (aluminum), the grounded enclosure (aluminum), and the SF_6_ gas region. The central conductor was assigned the operating voltage of 110 kV (phase-to-ground) for rated conditions, corresponding to 110 kV/√3 for phase-to-ground voltage in a 110 kV system, while the enclosure was set to ground (0 V). For lightning impulse conditions, a voltage of 550 kV was applied to the central conductor to evaluate the electric field distribution under extreme overvoltage scenarios [13]. The relative permittivity values used in the simulation were εr = 3.8 for the PA6 insulator material, and the aluminum electrodes were modeled as perfect electric conductors (PEC). The mesh consisted of approximately 50,000 triangular elements with local refinement in regions of high electric field gradient, particularly near the triple junctions and curved surfaces where field enhancement is expected. All external boundaries were set to electric insulation (n·D = 0) except where voltages were explicitly prescribed.

## 3. Results and Analysis

### 3.1. Analysis of Finished Product Quality Under Varying Melt Temperatures

Following the parameter settings described above, for the melt temperature variation study, the mold temperature was held constant at 120 °C for PET and 90 °C for PA6, while the melt temperature was incrementally increased at intervals of 10 °C. Simulations were conducted using the filling–packing–warping analysis sequence.

**Figure 2:**
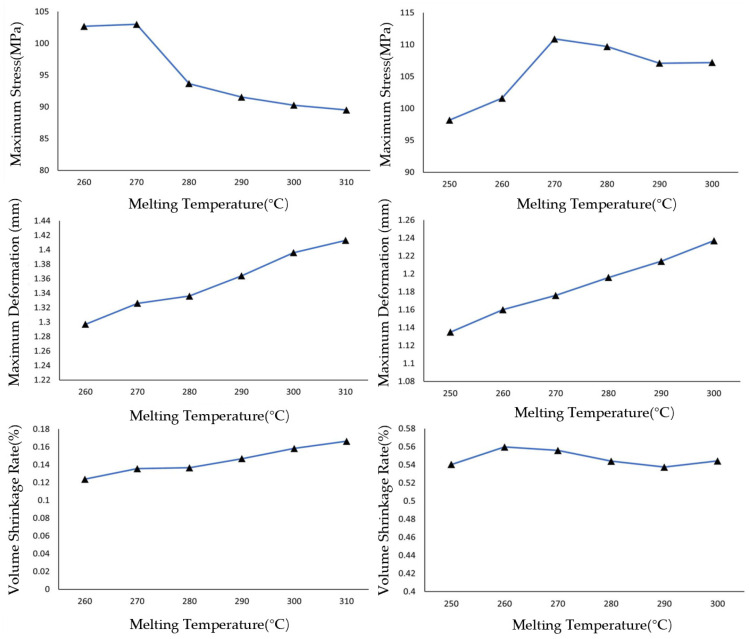
Variation in stress (**top**), deformation (**middle**), and shrinkage rate (**bottom**) for PET (**left**) and PA6 (**right**) with melt temperature changes at constant mold temperatures (PET: 120 °C; PA6: 90 °C).

As shown in Figure 2, with the melt temperature increasing at intervals of 10 °C, the maximum residual stress in both PET and PA6 injection-molded parts exhibits an initial rise followed by a decline, with peak values occurring near 270 °C for both materials. The variation range of residual stress is 12.8 MPa, corresponding to a 12.4% relative change for PET, and 12.1 MPa, corresponding to an 11.1% relative change for PA6. The deformation of both materials shows a steady upward trend with increasing melt temperature, with PA6 displaying slightly less deformation than PET. The analysis of finished product quality under varying melt temperatures concludes that the influence of melt temperature on key parameters follows the order: residual stress > deformation > volumetric shrinkage. The volumetric shrinkage of injection-molded parts is minimally affected by changes in melt temperature. Similarly, simulations were conducted by holding the melt temperature constant while incrementally increasing the mold temperature at intervals of 10 °C.

**Figure 3:**
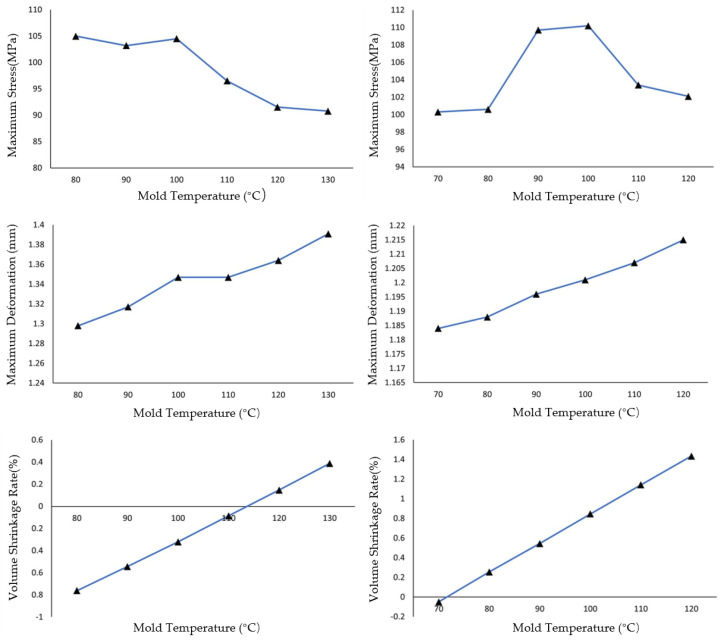
Variation in stress (**top**), deformation (**middle**), and shrinkage rate (**bottom**) for PET (**left**) and PA6 (**right**) with mold temperature changes at constant melt temperatures (PET: 290 °C; PA6: 280 °C).

As shown in Figure 3, as the mold temperature increases from 80 °C to 130 °C, the maximum residual stress of PET exhibits a pattern of initial fluctuation followed by a continuous decline: in the range of 80–100 °C, the stress remains between 103.5 and −105.0 MPa; above 100 °C, the stress continuously decreases from 104.5 MPa at 100 °C to 91.0 MPa at 130 °C. The maximum residual stress of PA6 exhibits an initial increase followed by a decrease: in the range of 70–100 °C, the stress continuously rises, reaching a peak of 111 MPa at 100 °C; above 100 °C, the stress continuously decreases to 102 MPa at 130 °C. The deformation of both materials continuously increases with higher mold temperature, with PA6 deformation being more sensitive to temperature variations; The volumetric shrinkage of both PET and PA6 increases nearly linearly with rising mold temperature, indicating that mold temperature is a key process parameter controlling the volumetric shrinkage of injection-molded parts. Within the same temperature variation range (from 80 °C to 130 °C for PET and from 70 °C to 120 °C for PA6), PA6 shows a greater change in volumetric shrinkage than PET. Quantitatively, the average shrinkage–temperature slope for PA6 is approximately 0.030%/°C, compared to 0.023%/°C for PET. This represents a 30% higher sensitivity for PA6. In terms of total change, the volumetric shrinkage of PA6 increases by 1.48% across its investigated mold temperature range, while PET increases by 1.15% across its range, making the absolute change in PA6 approximately 29% larger than that of PET.

This quantitative difference indicates that the thermal shrinkage behavior of PA6 is more sensitive to mold temperature variations than that of PET. The analysis of finished product quality under varying mold temperatures concludes that as the mold temperature increases, the volumetric shrinkage of injection-molded parts exhibits an approximately linear increase. The extent of influence of mold temperature on the three parameters can be ranked roughly as follows: volumetric shrinkage > residual stress > deformation. This ranking reflects the different sensitivities of these parameters to mold temperature variations, as discussed below. Volumetric shrinkage shows the largest absolute and relative changes (PET: 1.15%, 150.9%; PA6: 1.48%, 103.4%), indicating that it is most directly influenced by mold temperature. Residual stress exhibits moderate changes (PET: 14.0 MPa, 13.1%; PA6: 10.2 MPa, 9.3%), while deformation shows the smallest changes, suggesting that it is least sensitive to mold temperature variations among the three parameters.

Through comparison, it can be observed that residual stress is influenced almost equally by melt temperature and mold temperature, and the effect is significant. Deformation is also affected by both melt and mold temperatures, but to a lesser extent compared to residual stress. In contrast, volumetric shrinkage is almost exclusively directly influenced by mold temperature. By comparing the performance of PET and PA6 across different simulation parameter settings in terms of the three key parameters, it is observed that both materials exhibit roughly similar levels of residual stress. However, PET can achieve a slightly lower minimum stress than PA6 through optimization. In terms of deformation, PET and PA6 also demonstrate comparable performance. Regarding volumetric shrinkage, PA6 is more sensitive to changes in mold temperature compared to PET, exhibiting a larger variation range in volumetric shrinkage.

Based on the above analysis, an optimization function was developed to comprehensively evaluate the combined effect of the three key quality indicators. For a given set of parameters, the deviation from the minimum achievable value of each indicator (across all simulated parameter combinations for that material) is denoted as Δp (MPa) for residual stress, Δx (mm) for deformation, and Δs (%) for volumetric shrinkage.

The optimization objective function is defined as: Y = Δp/20 + Δx/0.5 + Δs/1.8. The denominators (20, 0.5, and 1.8) represent the typical variation ranges of each parameter based on the simulation results. The absolute and relative changes in each parameter with melt temperature are summarized in Table 4, while those with mold temperature are summarized in Table 5. As shown in these tables, residual stress (Δp) varies by approximately 15–20 MPa across the parameter space (PET: 12.8–14.0 MPa, PA6: 10.2–12.1 MPa), deformation (Δx) varies by approximately 0.5 mm (PET: 0.093–0.116 mm, PA6: 0.031–0.102 mm), and volumetric shrinkage (Δs) varies by approximately 1.8% (PET: 0.043–1.15%, PA6: 0.022–1.48%). By normalizing each deviation by its typical variation range, the three independent quality indicators are scaled to comparable magnitudes, allowing them to be combined into a single objective function. This prevents any single parameter from dominating the optimization due to differences in units or absolute magnitudes.

The additive linear form was chosen for its simplicity and interpretability, as it allows for the direct assessment of each parameter’s contribution to the overall quality index. This approach is commonly used in multi-objective engineering optimization, where multiple quality indicators must be balanced. This form assumes that the three quality indicators are independent and that their effects on overall part quality are approximately additive, which is reasonable given that they represent distinct physical phenomena (mechanical stress, geometric accuracy, and dimensional stability). Multiplicative forms were considered but deemed unsuitable because: (1) different units cannot be meaningfully multiplied, (2) a zero value for any single indicator would force the entire function to zero, which is overly restrictive, and (3) multiplicative forms do not align with the engineering intuition that quality losses from independent factors should be additive.

To verify the robustness of the weight selection, a sensitivity analysis was performed by varying each weight by ±20% and re-evaluating the optimal parameter combinations. The results showed that varying the weights by ±20% changed the optimal parameter selection by less than one temperature increment (10 °C), indicating that the optimization is robust to reasonable variations in weight selection.

Based on the above objective function, the optimal process parameters for the two materials are determined as follows:

PET: Melt temperature of 290 °C, mold temperature of 120 °C.

PA6: Melt temperature of 250 °C, mold temperature of 70 °C.

These optimal parameters correspond to the minimum values of the objective function Y across all simulated parameter combinations. Both optimal parameter sets fall within the extended parameter ranges studied: PET 290 °C melt (260–310 °C) and 120 °C mold (80–130 °C); PA6 250 °C melt (250–300 °C) and 70 °C mold (70–120 °C). Compared to the recommended processing ranges in the material datasheets, PET’s mold temperature (120 °C) is slightly above the recommended 100–110 °C, while PA6’s melt temperature (250 °C) and mold temperature (70 °C) are slightly below the recommended ranges (270–290 °C melt, 80–90 °C mold). These small deviations are within the extended parameter space investigated and are considered acceptable for the purposes of this study.

### 3.2. Coupled Simulation Analysis of Hydraulic Pressure Testing

Under a hydraulic pressure of 2.4 MPa without considering residual stress from the injection molding process, the stress results at the insert contact surface and within the bulk of both PET and PA6 are significantly below the compliance standard, with both materials exhibiting comparable stress levels.

As shown in Table 6, the stress levels of both materials are comparable. For hydraulic pressure testing at 2.4 MPa [12], the typical compliance requirements specify that the insert contact surface stress should not exceed 20 MPa and the bulk surface stress should not exceed 70–90 MPa. The simulation results for both PET (contact: 8.81 MPa, bulk: 19.04 MPa) and PA6 (contact: 9.12 MPa, bulk: 19.57 MPa) are significantly below these thresholds. This indicates that, in the absence of residual stress, both materials would meet the requirements of the hydraulic pressure test.

**Figure 4:**
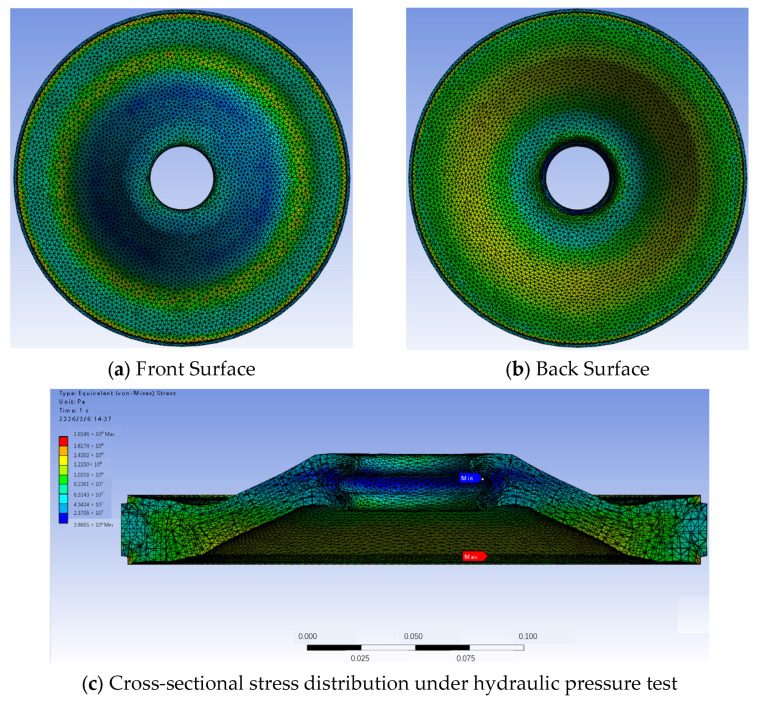
Surface stress distribution under hydraulic pressure test.

Taking the von Mises equivalent stress as the key indicator, the simulation shows that the pressure distribution characteristics of the injection-molded parts under different process parameters are generally consistent for both materials. The stress concentration occurs primarily at the inclined boundary region of the insulator, while the overall surface stress distribution remains relatively uniform, as illustrated in Figure 4.

As shown in Table 7, referring to the compliance criteria for hydraulic pressure testing (insert contact surface stress ≤ 20 MPa, bulk surface stress ≤ 70–90 MPa), the surface stress of the injection-molded part’s bulk ranges between 70 MPa and 90 MPa. When adopting a higher tolerance limit of 90 MPa, it meets the compliance requirements of the hydraulic pressure test. For PET material, under all parameter conditions, the insert contact surface stress ranges from 24.25 MPa to 27.55 MPa, with all values exceeding the 20 MPa compliance threshold. This indicates that PET injection-molded parts fail to pass the 2.4 MPa hydraulic pressure test under all parameter combinations investigated. In contrast, PA6 material exhibits insert contact surface stresses ranging from 18.29 MPa to 23.36 MPa. The minimum contact stress (18.29 MPa) falls below the 20 MPa compliance threshold, while the maximum contact stress (23.36 MPa) exceeds it. This indicates that under certain parameter combinations, PA6 can meet the hydraulic pressure test requirements.

**Figure 5:**
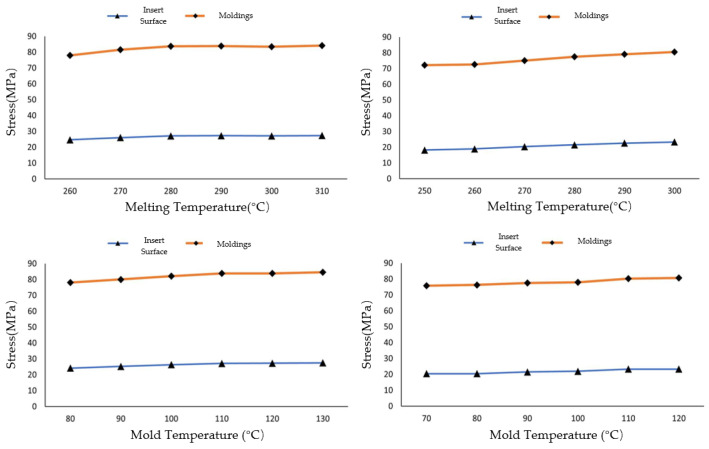
Stress variation in PET (**left**) and PA6 (**right**) with melt temperature (**top**) and mold temperature (**bottom**).

Specifically, based on the detailed simulation results shown in Figure 5, PA6 meets the compliance requirements when the melt temperature is below 270 °C and mold temperature is below 120 °C. Under these conditions, the insert contact surface stress remains below the 20 MPa threshold. For example, at the optimal processing parameters identified in Section 3.1 (melt temperature 250 °C, mold temperature 70 °C), the contact stress is approximately 18.5 MPa, well within the compliance limit. In contrast, parameter combinations with higher temperatures, such as melt temperature > 270 °C or mold temperature > 120 °C, result in contact stresses exceeding 20 MPa, with the maximum value of 23.36 MPa observed at the highest temperature combinations.

As shown in Figure 5, according to the simulation results, when PA6 material is processed with a melt temperature < 270 °C and a mold temperature < 120 °C, the resulting injection-molded part can pass the 2.4 MPa hydraulic pressure test. Therefore, although PA6 did not exhibit significant differences compared to PET in the simulation of the injection molding process, it demonstrates clear advantages over PET in terms of process performance during hydraulic pressure testing. Compared with the simulation results without applying initial stress, the coupled simulation results with initial stress show significantly higher stress levels. This indicates that the residual stress from the injection molding process plays a crucial role in determining the performance of the workpiece during hydraulic pressure testing.

### 3.3. Electric Field Analysis and Morphology Design of Insulators

For surfaces directly exposed to SF_6_ gas, which are susceptible to damage under extreme voltage impulses, it is essential to study their electric field distribution under lightning impulse voltage. Regarding the interior of the insulating component and the embedded parts, greater attention should be paid to the internal electric field distribution under operating conditions [11].

In the morphological design of insulators, a two-dimensional axisymmetric model is established for typical insulator application scenarios in GIS/GIL. The simulation domain includes the insulator, SF_6_ gas region, central high-voltage conductor (aluminum), and grounded enclosure (aluminum). The central conductor is assigned the operating voltage of 110 kV for rated conditions, while the enclosure is set to ground (0 V). For lightning impulse conditions, a voltage of 550 kV is applied to the central conductor [14].

The relative permittivity values used in the simulation are based on material properties and operating conditions: for PA6 insulator material, εr = 3.8 (from material datasheet); for SF_6_ gas at 0.4 MPa pressure, εr = 1.002; the aluminum electrodes were modeled as perfect electric conductors (PEC) [14]. The gas pressure of 0.4 MPa represents typical operating conditions in GIS/GIL systems.

The mesh consists of approximately 50,000 triangular elements with refinement in regions of high electric field gradient, particularly near the triple junctions and curved surfaces. Electrostatic field simulations are performed using COMSOL Multiphysics.

**Figure 6:**
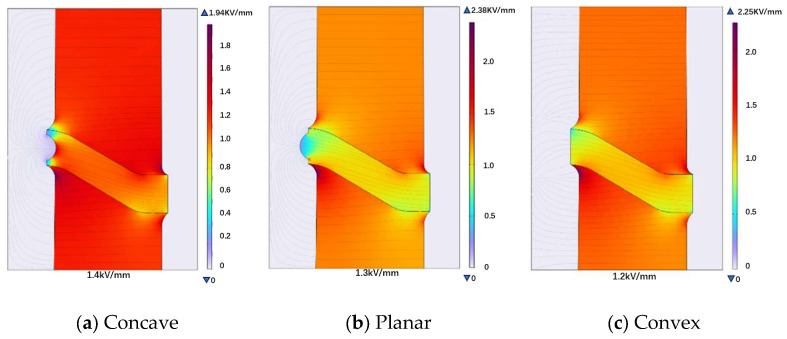
Electric field distribution under different insert contact surface designs. The maximum electric field strengths inside the insulator are 1.4 kV/mm, 1.3 kV/mm, and 1.2 kV/mm, respectively, as indicated by the circled regions.

Three different insulator–insert contact configurations are designed for electric field simulation, as shown in Figure 6. The three configurations represent typical interface geometries used in basin-type insulators, namely concave, planar, and convex contact surfaces. Both the concave surface (Figure 6a) and the planar surface (Figure 6b) feature a consistent curvature radius of 16.5 mm.

Simulation results indicate that changes in the insert contact surface design primarily affect the electric field strength inside the insulator, while having a relatively minor impact on the electric field within the gas. Among the three designs (concave, planar, and convex contact surfaces), the maximum electric field strengths inside the insulator are 1.4 kV/mm, 1.3 kV/mm, and 1.2 kV/mm, respectively (as shown in Figure 6). The convex contact surface achieves the lowest maximum internal electric field, indicating the most favorable field distribution, while the planar design exhibits the most uniform field distribution along the interface. All three designs meet the insulation design standards for internal fields under rated operating voltage (3 kV/mm).

Based on the insulation design standard of 3 kV/mm for internal insulator fields under rated operating voltage [14], the maximum withstand rated operating voltage for each design can be calculated using linear scaling, since the electric field is proportional to the applied voltage under electrostatic conditions. The calculation is as follows:

For the concave design: 110 kV × (3.0 kV/mm/1.4 kV/mm) ≈ 240 kV

For the planar design: 110 kV × (3.0 kV/mm/1.3 kV/mm) ≈ 255 kV

For the convex design: 110 kV × (3.0 kV/mm/1.2 kV/mm) = 275 kV

**Figure 7:**
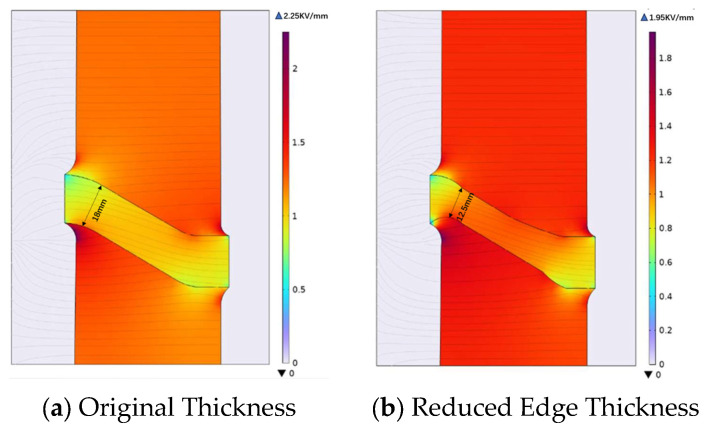
Electric field distribution of insulators with different edge thicknesses.

Among the three designs, the convex contact surface exhibits the lowest maximum electric field inside the insulator, indicating a more favorable electric field distribution., while the convex design enables the highest rated operating voltage. Considering assembly and fixation from a component integration perspective, designing a flat contact surface for the insert may pose challenges in assembly and securing. Therefore, adopting a convex design is beneficial as it facilitates fixation while controlling the electric field strength inside the insulator, thereby enabling a higher rated operating voltage. The simulation was performed with adjustments to the thickness of the insulator near its outer edge, as shown in Figure 7.

In this study, the edge thickness was reduced from 18 mm to 12.5 mm near the outer rim of the insulator, corresponding to a reduction of 5.5 mm (approximately 30.6%). This modification was chosen to evaluate the influence of edge thickness variations on electric field distribution.

As the edge thickness decreases, both the internal electric field of the insulator and the surface electric field exhibit an increasing trend, rising from approximately 2.1 kV/mm to 2.5 kV/mm (a 19% increase), while the maximum electric field on the housing surface shows a decreasing trend, falling from approximately 2.25 kV/mm to 1.95 kV/mm (a 13% decrease). These numerical values provide quantitative evidence for the observed trends.

The insulator’s ability to withstand lightning impulse is primarily constrained by the electric field intensity on the housing surface. Therefore, when necessary, appropriately reducing the edge thickness of the insulator can help reduce the electric field intensity on the housing surface by up to 13%, thereby enhancing the system’s ability to withstand lightning impulse conditions. However, this modification is also constrained by the 19% increase in internal electric field intensity, requiring a trade-off between this ability under the rated operating voltage and the lightning impulse voltage. For the original insulator design in this study, under the design requirements of a rated operating voltage of 110 kV and a lightning impulse voltage of 550 kV, the corresponding insulation standards can be met.

## 4. Conclusions

The main conclusions of this study are summarized as follows: (1) Regarding the injection molding process, this study investigated the effects of melt temperature and mold temperature on the quality of molded parts for both PET and PA6 through Moldflow simulation. The results show that, under variations in both melt temperature and mold temperature, residual stress is the most critical factor affecting the quality of injection-molded parts. By comprehensively analyzing the three core parameters, namely residual stress, maximum deformation, and volumetric shrinkage, this study identified the optimal processing parameters for the two materials using the objective function Y=Δp/20+Δx/0.5+Δs/1.8. The optimal processing parameters are 290 °C melt temperature and 120 °C mold temperature for PET, and 250 °C melt temperature and 70 °C mold temperature for PA6. These findings provide an important reference for the subsequent trial production of thermoplastic insulators. (2) Regarding hydraulic pressure testing, this study employed Moldflow–ANSYS coupled simulation to investigate the performance of injection-molded parts under hydraulic pressure tests across different process parameters. The results show that, among the two evaluation parameters, namely the bulk stress of the injection-molded part and the stress at the insert contact surface, the latter is the primary factor limiting compliance in hydraulic pressure testing. PET injection-molded parts failed to pass the 2.4 MPa hydraulic pressure test under all investigated parameter combinations, with insert contact surface stresses ranging from 24.25 to 27.55 MPa, all exceeding the 20 MPa compliance threshold. In contrast, PA6 can satisfy the hydraulic pressure test requirement within a processing window defined by melt temperature below 270 °C and mold temperature below 120 °C. These results highlight the potential advantages of PA6 over PET as an insulator material. (3) In terms of insulator morphology design, this study employed COMSOL to conduct static simulations of the electric field distribution in GIS/GIL insulators. Optimization recommendations were proposed for the design of the contact surface between the insulator and the insert. Based on the insulation design requirements within the SF6 system, this study also investigated the effect of changes in insulator edge thickness on electric field distribution. The results indicate that the convex contact surface design provides the most favorable internal electric field distribution among the investigated configurations. In addition, reducing the edge thickness of the insulator can reduce the electric field intensity on the housing surface under lightning impulse conditions, although this also increases the internal electric field intensity. Therefore, the structural design of thermoplastic insulators should balance the requirements of rated operating voltage and lightning impulse withstand performance. Overall, the results show that material selection, processing parameters, and morphological design should be considered together to achieve a thermoplastic insulator that simultaneously meets manufacturability, mechanical integrity, and electrical performance requirements.

This study has several limitations. First, as a simulation-only study, the results lack experimental validation through physical injection molding trials. Second, microstructural features such as fiber orientation distribution were not explicitly modeled, which may affect the accuracy of predicted mechanical and thermal properties. Third, the hygroscopic nature of PA6 was not considered, although moisture absorption may alter its dimensional stability and mechanical behavior under service conditions. Fourth, this study focused primarily on melt and mold temperatures, while other parameters such as injection speed and cooling rate distribution may also provide additional opportunities for optimization. Future work should therefore include experimental validation, incorporate fiber orientation effects and hygroscopic behavior through multi-physics simulations, and integrate insulator morphology optimization with the injection molding process for a more comprehensive design framework.

## Figures and Tables

**Table 1:** Comparison of the electrical properties of each material (30% glass fiber reinforcement).

Electrical Parameter	Epoxy Resin	PET (GF)	PA6 (GF)
Dielectric Strength (kV/mm)	31	36	39
Relative Permittivity	2.5~4.5	3.7	3.6
Dielectric Loss Factor (tanδ)	0.01	0.005	0.003
Volume Resistivity (Ω·m)	10^16^	10^15^	10^13^

**Table 2:** Comparison of thermal properties of each material (30% glass fiber reinforcement).

Thermal Parameter	Epoxy Resin	PET (GF)	PA6 (GF)
Glass Transition Temp. (°C)	105	80	55
Heat Deflection Temp. (°C)	130	213	210

**Table 3:** Values orranges of simulation process parameters.

Process Parameter	PET	PA6
Melt Temperature (°C)	260~310	250~300
Mold Temperature (°C)	80~130	70~120
Injection Pressure (MPa)	120	120
Injection Time (s)	20	20
Holding Pressure (MPa)	80	80
Holding Time (s)	90	90
Cooling Time (s)	120	120

**Table 4:** Absolute and relative changes in each parameter with melt temperature.

Parameter	PET (Absolute)	PET (Relative)	PA6 (Absolute)	PA6 (Relative)
Residual Stress	12.8 MPa	12.4%	12.1 MPa	11.1%
Deformation	0.116 mm	8.2%	0.102 mm	8.2%
Volumetric Shrinkage	0.043%	25.6%	0.022%	4.0%

**Table 5:** Absolute and relative changes in each parameter with mold temperature.

Parameter	PET (Absolute)	PET (Relative)	PA6 (Absolute)	PA6 (Relative)
Residual Stress	14.0 MPa	13.1%	10.2 MPa	9.3%
Deformation	0.093 mm	6.7%	0.031 mm	2.6%
Volumetric Shrinkage	1.15%	150.9%	1.48%	103.4%

**Table 6:** Hydraulic pressure simulation results without initial stress.

Material	PET	PA6
Insert Contact Stress (MPa)	8.81	9.12
Bulk Stress (MPa)	19.04	19.57

**Table 7:** Summary of hydraulic pressure test performance with applied initial stress.

Material	PET	PA6
Max Bulk Stress (MPa)	84.52	80.68
Min Bulk Stress (MPa)	78.03	72.26
Min Contact Stress (MPa)	24.25	18.29
Max Contact Stress (MPa)	27.55	23.36

## Data Availability

The original contributions presented in this study are included in the article. Further inquiries can be directed to the corresponding author.
